# Bacteraemia in Intensive Care Unit: Clinical, Bacteriological, and Prognostic Prospective Study

**DOI:** 10.1155/2017/4082938

**Published:** 2017-03-19

**Authors:** Zineb Lachhab, Mohammed Frikh, Adil Maleb, Jalal Kasouati, Nouafal Doghmi, Yassine Ben Lahlou, Bouchra Belefquih, Abdelhay Lemnouer, Mostafa Elouennass

**Affiliations:** ^1^Bacteriology Department, Mohammed V Military Teaching Hospital, Rabat, Morocco; ^2^Research Team, Bacterial Epidemiology and Resistance, Mohammed V University, Faculty of Medicine and Pharmacy, Rabat, Morocco; ^3^Microbiology Department, Mohammed VI University Hospital, Oujda, Morocco; ^4^Epidemiology Department, Mohammed V Military Teaching Hospital, Rabat, Morocco; ^5^Medical Reanimation Department, Mohammed V Military Teaching Hospital, Rabat, Morocco

## Abstract

*Objectives.* We conducted a one-year observational study from December 2012 to November 2013 to describe the epidemiology of bacteraemia in intensive care units (ICU) of Mohammed V Military Teaching Hospital of Rabat (Morocco).* Methods.* The study consisted of monitoring all blood cultures coming from intensive care units and studying the bacteriological profile of positive blood cultures as well as their clinical significance.* Results.* During this period, a total of 46 episodes of bacteraemia occurred, which corresponds to a rate of 15,4/1000 patients. The rate of nosocomial infections was 97% versus 3% for community infections. The most common source of bacteraemia was the lungs in 33%, but no source was identified in 52% of the episodes. Gram negative organisms were isolated in 83,6% of the cases with* Acinetobacter baumannii* being the most frequent. Antibiotic resistance was very high with 42,5% of extended-spectrum beta-lactamases (ESBLs) in Enterobacteriaceae and 100% of carbapenemase in* Acinetobacter baumannii*. The antibiotherapy introduced in the first 24 hours was adequate in 72% of the cases.* Conclusions.* Bloodstream infections in ICU occur most often in patients over 55 years, with hypertension and diabetes. The bacteria involved are mainly Gram negative bacteria multiresistant to antibiotics. Early administration of antibiotics significantly reduces patients mortality.

## 1. Background

Bacteraemia continues to be an important cause of morbidity and mortality despite the availability of potent antimicrobial agents and sophisticated diagnostic means [1]. There is a continuous rise worldwide in bacteraemia incidence, which is related to many factors as the population demographic differences and risk factor distribution in regions [2].

Over the past 30 years, the incidence, etiology, and epidemiology of bacteraemia have changed with the evolution of medical care [3]. In developed countries, these parameters are well known through regular monitoring [4]. Thus, several studies have treated the etiologic agents most frequently responsible for these infections and improved the understanding of the risks and consequences associated with them [5]. In resource-limited countries, such as Morocco, the lack of sufficient data makes bacteraemia one of the daily worries of medical practice especially in intensive care units (ICU) where patients are predisposed to acquire them.

Bacteraemia is a major problem to manage. It is indeed necessary to find a compromise between the urgency of the treatment and the difficulty to pose a precise diagnosis within a short time [1]. In this context, it is important for the clinician to know the most frequently encountered bacterial species and their sensitivity to antibiotics to initiate prompt and effective empiric antibiotic therapy. As a part of the evolution of the bacterial ecology and the changes in the levels of resistance to antibiotics, periodic revaluation of the management of bacteraemic patients is necessary, in order to tailor for better treatment decisions [6].

The aim of this study is to determine the incidence of bacteraemia in ICU of our hospital, to establish the bacterial epidemiology and the susceptibility of the isolated strains to antibiotics.

## 2. Methods

This observational study was conducted prospectively at the medical and surgical ICUs of Mohammed V Military Teaching Hospital of Rabat, Morocco. The study was designed to include all sequentially encountered episodes of bacteraemia during the period December 2012–November 2013.

The study consisted of monitoring, from the bacteriology laboratory, all blood cultures coming from ICU. Aerobic and anaerobic blood culture vials were performed at the patient's bed by peripheral puncture or through a peripheral or central device newly installed. They are transported then to the bacteriology laboratory where they are incubated with agitation at 37°C in the system Bactec 9240 (Becton Dickinson).

We perform, from positive bottles, a transplanting on enriched medium and smear for Gram stain. Based on this direct examination and the front of a monomorphic appearance, biochemical identification and an antibiogram were made directly from the blood culture broth. The identification of species was based on the growth and the morphological and biochemical characteristics (API gallery, Biomérieux, Marcy-Star/France). The antimicrobial susceptibility testing of the isolates was performed by disk diffusion agar method, as recommended by the Committee of the Susceptibility of the French Society for Microbiology [7].

Each positive blood culture leads to a clinical survey by the author and the filling of an exploitation plug [8]. Concerning antibiotic treatment, we retained an average length of 10 days of treatment; and we noted the treatment received by the patient from day 0 of bacteraemia (the day of collection of blood culture) to day 10. The cost of antimicrobial treatment per day per infected patient was calculated on the basis of the antimicrobial agent initiated on day 0 (including inappropriate treatment) and by multiplying the unit's price per dose and per the number of daily doses up to day 10. The costs are calculated in Moroccan Dirham (MAD) and expressed in Euro (EUR/MAD = 10), on the basis of the 2012 price of antimicrobial agents provided by the military hospital's pharmacy.

Quantitative variables were expressed as mean +/− standard deviation. Chi square test was used to compare data between different groups; the results were considered significant if the *p* value was less than 0.05. Statistical analysis was carried out using the software package SPSS v10.0.

## 3. Results

From December 2012 to November 2013, 46 episodes of bacteraemia were recorded in 39 patients; the overall incidence was 20/1000 days of hospitalization and 15.4/1000 patients admitted at ICU.  Table 1 shows the demographic and clinical data of patients. Most patients (97%) had nosocomial bacteraemia and primary bacteraemia accounted for 26.7% of the cases. The most common source of bacteraemia was the lungs (33%) but no source was identified in 52% of cases (Table 2). There was 83.6% Gram negative bacilli (GNB) bacteraemia; overall,* Acinetobacter baumannii* was the most offending germ in bacteraemia and* Klebsiella pneumoniae* in contamination. Regarding yeasts, one germ out of the four isolated was responsible for bacteraemia; it was of* Candida tropicalis*. Commensal bacteria were responsible for contamination in all the cases (70.5%) (Table 2).

Thirty-eight (69%) of bacteraemic episodes were caused by antibiotic-resistant organisms. Among the GNB, all* Pseudomonas *spp. and* Acinetobacter *spp. were sensitive to colistin; however, a resistance to imipenem was observed among total isolates of* Acinetobacter* (13/13) in the context of the production of carbapenemase, while* Pseudomonas aeruginosa* resistance is emerging with a rate of 20%. The rate of Enterobacteriaceae with ESBL in this study was 42.5% essentially present in* Klebsiella pneumoniae* with a rate of 44%. Only 20% (1/5) of* Staphylococcus aureus* isolates were resistant to methicillin; and on the other hand, no Gram positive germ showed decreased susceptibility to glycopeptides (Table 3).

**Figure 1:**
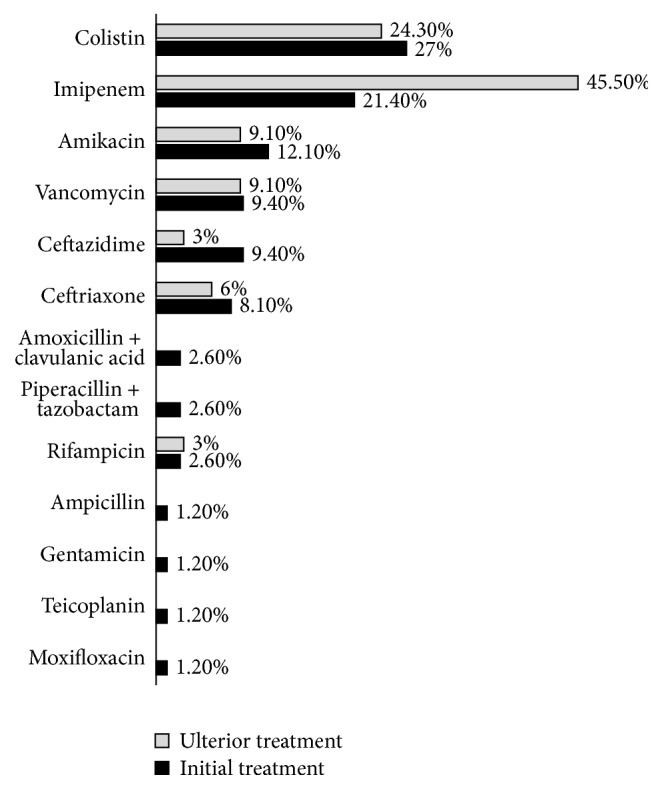
Molecules prescribed for initial and ulterior antibiotic therapy.

With regard to antibiotic therapy, we found that almost all patients (97.5%) received a curative antibiotic therapy that has proven adequate in the first 24 hours of its introduction in 72% of the cases, but the rate of efficiency was limited to 37%. This initial treatment was an association of antibiotics in 57% of the cases. The most frequent association was beta-lactam with aminoglycosides, while the most used drug in monotherapy was colistin and imipenem (21.4%). The molecules used are indicated in Figure 1. Initial treatment lasted in 33% of the cases one day before being changed; the main reasons for this change were either being inadequate, being ineffective, or realization of deescalation. However, ulterior treatment was documented in 59% of cases and remained probabilistic in 41% of cases. The antibiotic association rate was higher than the initial treatment, with a value of 82%. The most common association was the beta-lactams and polymyxins, while imipenem was the most prescribed molecule (Figure 1). This treatment proved adequate in 93% of the cases and effective in 65% of the cases (Table 4).

Twenty-seven patients (69%) died during their ICU stay. For 21 of them, the death was directly attributed to bacteraemia. Death rate in the population under study compared to all deaths in the ICU during the study period was 12.5%. The delay between the occurrence of bacteraemia and death was on average of 8,4 days. Comparisons of demographic and clinical characteristics of survived and deceased patients are described in Table 5.

## 4. Discussion

The incidence of bacteraemia in our study was 15.4/1000 patients. Several reasons can explain the discrepancy of the incidence rate between our study and the other international studies, such as the type of the studied population and blood culture rates [9, 10].

Our demographics data showed that bacteraemia affects mainly males and elderly population (>55 years), in agreement with several studies [1, 11, 12]. The predominance of men appears without explanation, unlike the advanced age which is related to a deterioration of the immune system exposing the elderly to a high risk of contracting infectious diseases including bacteraemia. Zhang et al. reported that catheterization, mechanical ventilation, and urinary catheter were considered as risk factors for bacteraemia acquisition [13]. Our study supported the correlation of this factors and the high incidence found, since almost all of our population has been exposed to such maneuvers.

In this study, we confirm the predominance of GNB bacteraemia since we found it at the rate of 83.6%; this theory has also been found in a recently published international observational study providing information about bacteraemia from 1,156 ICU patients worldwide [14]. The most alarming GNB in our case was* Acinetobacter baumannii* with a frequency of 23.6%; this result appears wary compared to those reported in the literature [15].* Acinetobacter baumannii* is responsible for more and more severe infections and the incidence of bloodstream infections caused by this organism is constantly increasing [16]. A study conducted in the UK showed that the rate of bacteraemia caused by* Acinetobacter *spp. increased from 2 to 18 per 100,000 hospital days between 1998 and 2005. Resistance to carbapenems has increased during the same period from 0% to 55% [17]. The carbapenems are the antibiotics of choice for the treatment of serious infections due to multidrug-resistant* Acinetobacter baumannii*. Unfortunately, the number of isolates of* Acinetobacter baumannii* resistant to carbapenems has increased in the recent years, which is a big problem because the resistance to carbapenems limits the clinician's options for successful treatment and leads to increased mortality [17]. Indeed the* Acinetobacter baumannii* isolates in our study showed 100% resistance to imipenem. This rate is extremely higher than that reported in the literature [18]. Several studies have indicated that risk factors for carbapenem-resistant* Acinetobacter baumannii* acquisition were higher in longer hospital stays, invasive procedures, admission to ICU, and irrational use of carbapenems [19, 20].

*Klebsiella pneumoniae* was the second organism causing bacteraemia in our study with a rate of 18.2%. This value is higher than that reported in other studies [1]. This germ presented a high resistance rate vis-a-vis the beta-lactams and aminoglycosides. ESBL rate in our study was very high compared to that reported in the study of Elouennass et al. [21] in 2008 that was conducted in the same structure as ours (18%). In general, Enterobacteriaceae isolated from the positive blood cultures, coming from the ICU during the period of study, showed a very high rate of resistance to third-generation cephalosporins related essentially to an ESBL phenotype present in 42.5% of all isolated strains of Enterobacteriaceae and touching in the first isolates of* Klebsiella pneumoniae*.* Pseudomonas aeruginosa* was isolated from 9.1% of bacteraemic episodes; this frequency joined the results of the literature [1, 10].

*Staphylococcus aureus* in this study represented 9.1% of bacteraemia. This rate is lower than the data reported by other studies [10] and this could be due to the low rate of catheter bloodstream infections in our study. MRSA rate was 20%, which is lower than the results reported by several studies [12, 19]. Among the Gram positive bacteria, we did not encounter any resistance to vancomycin or teicoplanin in our isolates; this result is completely different from several studies [12, 19]. We have detected four cases of positive blood cultures for* Candida*, and only one corresponded to bacteraemia. A low rate of positive blood cultures yeast was also reported in the study of Elouennass et al. [21]. We can retain as an explanation the fact that blood cultures performed on conventional aerobic bottle, all the more on anaerobic environment, are less efficient than blood cultures performed on Sabouraud medium [22].

The absence of coagulase negative staphylococci (CNS) bacteraemia in our study can be explained by the fact that the judgment criterion for the definition of these infections requires the presence of several positive blood cultures with the same antibiotype, which was missing in our case and this could be unnoticed true CNS bacteraemia. On the other hand, the most threatening germ in contamination was CNS; Martinez et al. reported that 95% of germs contaminating blood cultures drawn from central lines and 88% of germs contaminating blood taken from peripheral vein blood cultures are CNS [23].

The problem of subjectivity in the interpretation of a positive blood culture certainly plays a role in the difference of the results observed in the literature and it is thus necessary to recall the difficulty of establishing the clinical significance of low virulence isolates such as commensal flora.

The most frequent source of bacteraemia microbiologically identified was the respiratory system (33%) followed by urinary system (10%). This is in agreement with many reports [15]. In cases where no source has been determined, we suppose that undiagnosed vascular catheter infections or bacterial translocation from the gastrointestinal tract may explain the infection.

In terms of treatment, the initial treatment adequacy rate is the most discussed criterion in international journals because it influences the evolution of bacteraemic patients. Comparing our results to those in the literature, we noted that the adequacy rate of initial antibiotic therapy in our study is comparable to that reported by many studies [1, 10].

Colistin emerged in the last decade as a savior for the treatment of critically septic patients who suffer MDR-GNB infections [24]. It is frequently used as combination therapy in order to maximize killing effect of pathogens, reducing rate of resistance, optimizing clinical outcome, and reducing mortality [25]. However two earlier meta-analyses published in 2003 and 2004 in BMJ revealed that combination therapy did not change rates of fatality and increased adverse events [24].

In regard to our practice, colistin is usually used in combination with imipenem for the treatment of severe infections especially those due to* Acinetobacter baumannii*. It is used as monotherapy in the case of MDR* Acinetobacter baumannii* infections, taking into account the clinical condition and the evolution of the patient.

Pirson et al. studied the total cost of bacteraemia through a retrospective analysis of two cohorts totaling 1344 patients in a Belgian establishment in 2001. The authors concluded that bacteraemia was related to additional cost of € 12,853 per patient [26].

According to this study, we can confirm the additional cost associated with bacteraemia in terms of consumption of antibiotics, but the extent of this problem cannot be measured because of the existence of socioeconomic differences preventing us from comparing our results to those of Western studies on one hand and the lack of studies of the cost in the developing countries on the other hand.

Our study shows a death rate of 69%; this rate appears higher than those reported by several international studies [1, 10]. The heterogeneity of the studied populations, the endemic rate of multiresistant bacteria, the management difficulties, and the differences among the work designs followed by each team could be explanations for this difference.

Mortality related to bacteraemic episodes increases with age, especially for patients over 75 years old who are frequently hospitalized, are suffering from several diseases, and have a reduced immune status. Furthermore clinical presentation of bacteraemia in this population is sometimes atypical, delaying diagnosis [27]. In our study, a difference of 10-year average was noted between patients who died and those who survived in favor of the first. But this result appears to be not statistically significant; this requires the recruitment of a larger number of patients in future studies. Nosocomial bloodstream infections are known to have a higher attributable mortality than infections acquired outside the hospital; Garrouste-Orgeas and colleagues [28] found that nosocomial bacteraemia was associated with a threefold increase in mortality. In our case, we were not able to draw such a conclusion, since we have a single episode bacteraemic community. Mortality due to episodes of bacteraemia increases with the accumulation of predisposing conditions in the same patient. Indeed, the identification of underlying illness in patients admitted to ICU darkens the prognosis of bacteraemia [27]. The presence of an antecedent of hospitalization, corticosteroid therapy, antibiotic therapy, and surgery appear to be important risk factors of mortality. These criteria also stand out as mortality risk factors in the study of Kang et al. [29]. We observed a higher mortality rate when the source of infection was pulmonary; such observations are found in the literature [27]. Several studies have shown that mortality from bacteraemic episodes increases with inadequate treatment. Our numbers are too small to be interpreted. There are intuitively interests to start early empirical antibiotic that is active in vitro on the germ involved; this benefit has been demonstrated in several studies [30].

## 5. Conclusions

This study highlights the predominance of Gram negative bacteria and the emergence of multidrug-resistant organisms. Our results confirm some facts reported in many publications like the resistance to antibiotics and the attributable mortality; however, they differed in several important parameters like the clinical significance of positive blood cultures for CNS and the microbial ecology of bacteraemia. We also found that the rate of appropriate antibiotic treatment in the intensive care units of our structure is comparable to that reported by some international hospitals in big fame. The main limit of our study is the low number of patients; this instigates us to lead a multicentric study in order to provide a more complete picture of microbial ecology and resistance trends and to produce meaningful guidelines for bacteraemia prevention.

## Figures and Tables

**Table 1:** Demographic and clinical data of the 39 patients involved in the study.

Variable		Number, mean +/− SD	%
Gender	M	24	62%
	F	15	38%

Age (mean +/− SD)		59,1 +/− 7,6	—

Age group	15–25	2	5,1%
	25–35	2	5,1%
	35–45	2	5,1%
	45–55	4	10,2%
	55–65	14	35,9%
	65–75	11	28,2%
	75–85	4	10,2%

Underlying illness	Essential hypertension	13	34,2%
	Diabetes mellitus	12	30,8%
	Immunosuppression	3	8,1%
	Chronic renal disease	2	5,4%
	Ischemic heart disease	4	10,4%
	Others	12	31,6%

Severity	Sepsis	23	50%
	Severe sepsis	8	17,4%
	Septic shock	15	32,6%

Scores of gravity	GLASGOW II	10,14 +/− 3,76	—
	APACHE II	18,95 +/− 3,76	—
	Predicted mortality	35,07 +/− 22,2	—

Length of stay		41 +/− 6,4	—

Antecedents	Recent antibiotic use	13	33,3%
	Recent hospitalization	23	59%
	Recent surgery	14	34,5%
	Recent corticosteroid therapy	24	61,5%

Outcome	Clinical failure	17	44%
	Clinical success	22	56,5%

Bacteraemia acquisition time		16,2 +/− 20,27	—

**Table 2:** Distribution of different isolates in relation to setting of source of bacteraemia.

Families	Bacteria	Total (%)	Bacteraemia (%)	Contamination (%)	Source of bacteraemia			
					UN	P	U	CVC
Enterobacteriaceae	*E. aerogenes*	1 (0,7%)	1 (100%)	0 (0%)	0 (0%)	1 (100%)	0 (0%)	0 (0%)
	*E. cloacae*	3 (2%)	2 (67%)	1 (33%)	1 (50%)	0 (0%)	0 (0%)	1 (50%)
	*E. coli*	3 (2%)	2 (67%)	1 (33%)	1 (50%)	0 (0%)	1 (50%)	0 (0%)
	*K. pneumoniae*	16 (10,7%)	10 (62,5%)	6 (37,5)	9 (90%)	0 (0%)	1 (10%)	0 (0%)
	*M. morganii*	2 (1,3)	2 (100%)	0 (0%)	1 (50%)	0 (0%)	0 (0%)	1 (50%)
	*P. mirabilis*	5 (2,7)	2 (40%)	3 (60%)	2 (100%)	0 (0%)	0 (0%)	0 (0%)
	*P. stuartii*	2 (1,3)	1 (50%)	1 (50%)	1 (100%)	0 (0%)	0 (0%)	0 (0%)
	*S. marcescens*	3 (2%)	2 (67%)	1 (33%)	2 (100%)	0 (0%)	0 (0%)	0 (0%)
	*S. odorifera*	5 (3,3)	3 (60%)	2 (40%)	1 (33%)	2 (67%)	0 (0%)	0 (0%)

Nonfermentative Gram negative bacilli *n* = 20	*A. baumannii*	18 (12%)	13 (72%)	5 (28%)	4 (31%)	7 (54%)	0 (0%)	2 (15%)
	*P. aeruginosa*	10 (6,7)	5 (50%)	5 (50%)	3 (60%)	1 (20%)	1 (20%)	0 (0%)
	*Chryseobacterium *sp.	1 (0,7)	0 (0%)	1 (100%)	/	/	/	/
	*Sphingomonas *sp.	1 (0,7)	1 (100%)	0 (0%)	1 (100%)	0 (0%)	0 (0%)	0 (0%)
	*S. maltophilia*	2 (1,3)	1 (50%)	1 (50%)	1 (100%)	0 (0%)	0 (0%)	0 (0%)

Staphylococci *n* = 65 (44%)	*S. aureus*	5 (3,3%)	5 (100%)	0 (0%)	2 (40%)	3 (50%)	0 (0%)	0 (0%)
	*CNS*	60 (40%)	0 (0%)	40 (100%)	/	/	/	/

Streptococci *n* = 6 (4%)	*E. faecalis*	5 (3,3%)	2 (40%)	3 (60%)	2 (100%)	0 (0%)	0 (0%)	0 (0%)
	*S. mitis*	1 (0,7%)	1 (100%)	0 (0%)	1 (100%)	0 (0%)	0 (0%)	0 (0%)

Gram positive bacilli in *n* = 2 (1.3%)	*Bacillus *spp.	1 (0,7%)	0 (0%)	1 (100%)	/	/	/	/
	*Corynebacterium *sp.	1 (0,7%)	0 (0%)	1 (100%)	/	/	/	/

BGN demanding *n* = 1 (0.7%)	*H. influenza*	1 (0,7%)	1 (100%)	0 (0%)	0 (0%)	1 (100%)	0 (0%)	0 (0%)

Yeasts *n* = 4 (2.7%)	*C. albicans*	1 (0,7%)	0 (0%)	1 (100%)	/	/	/	/
	*C. non-albicans*	3 (2%)	1 (33%)	2 (67%)	0 (0%)	0 (0%)	1 (100%)	0 (0%)

UN: unknown, P: pulmonary, U: urinary, and CVC: central venous catheter.

**Table 3:** Antimicrobial resistance rate of Gram negative bloodstream isolates.

Drug	Enterobacteriaceae (*n* = 25)	*Klebsiella Pneumoniae *(*n* = 10)	Nonfermentative GNB (*n* = 20)	*Acinetobacter baumannii *(*n* = 13)	*Pseudomonas aeruginosa *(*n* = 5)
Amoxi-Clavulanate	64,2%	60%	NT	NT	NT
Pip-Tazobactam	23,8%	20%	82,3%	100%	20%
Cefalotin	64,2%	60%	NT	NT	NT
Ceftazidime	57,1%	60%	70%	100%	20%
Ceftriaxone	50%	60%	NT	NT	NT
Imipenem	0%	0%	78,9%	100%	20%
Ertapenem	12,5%	20%	NT	NT	NT
Cefoxitime	8,3%	10%	NT	NT	NT
Gentamicin	37,5%	50%	63,1%	83,3%	20%
Tobramycin	32%	50%	50%	53,8%	20%
Amikacin	4%	0%	47,3%	50%	20%
Netilmicin	36%	50%	45%	46,1%	40%
Ciprofloxacin	40%	70%	70%	100%	20%
Cotrimoxazole	64%	80%	85%	92,3%	100%
Fosfomycin	0%	0%	NT	NT	NT
Colistin	0%	0%	0%	0%	0%
Ticarcillin	NT	NT	88,2%	100%	33,3%
Piperacillin	NT	NT	72,2%	100%	20%
Rifampicin	NT	NT	42,8%	46,1%	0%

**Table 4:** Characteristics of antimicrobial treatment.

Characteristics	Initial treatment (*n* = 43)		Further treatment (*n* = 17)	
	Number	%	Number	%
(i) Delay of introduction:				
(a) <24 h	42	98%	—	—
(b) 24 h ≪ 48 h	0	0%	—	—
(c) >48 h	1	2%	—	—

(ii) Type of prescription:				
(a) Not specifically adapted	22	51%	—	—
(b) Probabilistic	20	46,5%	7	41%
(c) Documented	1	2,5%	10	59%

(iii) Type of treatment:				
(a) Monotherapy	18	43%	3	18%
(b) Association	25	57%	14	82%

(iv) Average length of initial treatment: (mean +/− SD)	4,2 +/− 0,6		—	

(v) Adequacy of treatment:				
(a) Adequate	31	72%	16	93
(b) Inadequate	12	28%	1	7%

(vi) Effectiveness:				
(a) Efficient	27	63%	6	35%
(b) Ineffective	16	37%	11	65%

(vii) Initial treatment modification reason:				
(a) Escalation	—	—	4	21%
(b) Deescalation	—	—	13	79%

(viii) Most prescribed family:				
(a) Beta-lactams	12	28%	3	18%
(b) Polymyxins	8	18,7%	—	—
(c) Beta-lactams + aminoglycoside	8	18,7%	1	6%
(d) Beta-lactams + polymyxins	6	14%	8	47%

(ix) The most commonly used antibiotics:				
(a) Imipenem	9	21,4%	8	45,5%
(b) Colistin	12	27%	4	23,3%
(c) Amikacin	28	12,1%	2	9,1%
(d) Vancomycin	22	9,4%	2	9,1%

(x) Cost of treatment:				
(a) Total cost per patients	899,27 Euros [259,85–1544,69]			
(b) Daily cost per patients	31,01 Euros [17,48–54,59]			

**Table 5:** Comparison between the dead and survived patients in the study based on demographic, clinical, and microbiological profiles.

Variable		Survived (*n* = 12)		Dead (*n* = 27)		Signification
		Number	%	Number	%	
Age (mean +/− SD)		52,1 +/− 20,1		62,3 +/− 12,5		NS

Gender	M	4	33%	11	40,7%	NS
	F	8	67%	16	59,2%	

Underlying illness	Diabetes mellitus	3	25%	9	33,3%	NS
	Essential hypertension	1	8,3%	10	37%	NS
	ID	1	8,3%	1	3,7%	NS
	Chronic renal diseases	0	0%	2	7,4%	NS
	Cardiopathy	1	8,3%	3	11,1%	NS

Origin of bacteremia	Nosocomial	12	100%	26	96,2%	NS
	Ambulatory	0	0%	1	3,7%	NS

Nature of bacteremia	Primary	7	58,3%	15	55,5%	NS
	Secondary	5	41.6%	12	44,4%	NS

Source of bacteraemia	Lungs	3	25%	10	37%	NS
	Urinary tract	2	16,6%	2	7,4%	NS
	CVC	1	8,3%	2	7,4%	NS
	Unknown	6	50%	13	48,1%	NS

Severity	Sepsis	9	75%	7	25,9%	0,007
	Severe sepsis	2	16,6%	4	14,8%	NS
	Septic shock	1	8,3%	16	59,2%	0,011

Type of stained bacteria	GNB	8	67%	25	92,5%	NS
	GPC	3	25%	2	7,4%	NS
	MDR	9	75%	17	62,9%	NS

Microorganism	*A. baumannii*	3	25%	10	37%	NS
	*K. pneumoniae*	3	25%	7	25,9%	NS
	*P. aeruginosa*	2	16,6%	3	11,1%	NS
	*S. aureus*	3	25%	2	7,4%	NS

Adequacy of treatment	Adequate	5	41.6%	18	66,6%	NS
	Inadequate	5	41.6%	9	33,3%	NS

## Abbreviations

ICU: Intensive care units
ESBL: Extended-spectrum beta-lactamases
APACHE II: Acute Physiology and Chronic Health Evaluation II
GNB: Gram negative bacilli
CNS: Coagulase negative staphylococci.
